# Association of objectively assessed physical activity with immune cell infiltration of the tumor microenvironment in non-metastatic colon cancer

**DOI:** 10.1007/s00384-026-05232-5

**Published:** 2026-09-10

**Authors:** David Renman, Sofia Edin, Ulf Gunnarsson, Niillas Blind, Karin Strigård, Richard Palmqvist

**Affiliations:** 1 Department of Diagnostics and Intervention, Umeå University, SE-901 88 Umeå, Sweden; 2 Department of Medical Biosciences, Pathology, Umeå University, Umeå, Sweden

**Keywords:** Accelerometer, Colon cancer, Immune infiltration, Physical activity, Physical fitness, Tumor microenvironment

## Abstract

**Purpose:**

Physical activity and immune cell infiltration of the tumor microenvironment are associated with improved prognosis in colon cancer. There is also evidence that physical activity may mobilize immune cells against colon tumors. One possible mechanism behind the positive effect of physical activity on colon cancer prognosis could thus be increased immune cell infiltration of the tumor microenvironment. There are few previous studies examining this association, and none has included an objective assessment of physical activity.

**Methods:**

This was a study on 69 patients enrolled in the Physical Activity and its impact on COlon cancer Surgery (PACOS) prospective cohort, all diagnosed with colon cancer stages I–III, all eligible for curative surgery, and with no previous treatment. Prior to surgery, the participants underwent physical tests, dual-energy X-ray absorptiometry, and wore an accelerometer to quantify their physical activity and physical fitness. Tumor infiltration by cytotoxic T cells, T-helper 1 cells, regulatory T cells, B cells, and macrophages was objectively assessed with multispectral quantitative automated pathology imaging. Odds ratios for associations between physical activity and immune cell infiltration were estimated.

**Results:**

A higher number of repetitions in the 30-s sit-to-stand test was associated with a lower density of T-helper 1 cells in the stromal compartment of the tumor, in the model adjusted for age and sex. When correcting for multiple testing, the association was considered non-significant. No other significant association was seen for any of the physical tests or immune cell types.

**Conclusions:**

No consistent evidence was found to support the hypothesis of an association between physical activity or fitness and increased immune cell tumor infiltration in colon cancer.

**Trial registration:**

This study was registered at clinicaltrials.gov (ID NCT03947840), registration date 2019-05-19.

**Supplementary Information:**

The online version contains supplementary material available at https://doi.org/10.1007/s00384-026-05232-5.

## Background

Colon cancer is one of the most prevalent malignancies worldwide representing 6% of newly diagnosed cancer cases and 5.8% of cancer deaths each year [1]. The risk of developing colon cancer is associated with several environmental risk factors. Consumption of processed meat, red meat, obesity, and alcohol consumption is associated with higher risk while physical activity and consumption of whole grains, dietary fibers, and dairy products are associated with lower risk [2]. Higher levels of physical activity both before and after diagnosis may also improve prognosis [3, 4].

Prognosis is also affected by immune cell infiltration of the tumor microenvironment with greater tumor lymphocyte infiltration associated with better prognosis [5]. The immune response in the tumor microenvironment is associated with prognosis independent of sex, age, tumor stage, node stage, and microsatellite instability [6]. In a recent meta-analysis of immune cell infiltration of the tumor microenvironment in colorectal cancer, high numbers of cell types analyzed (CD3, CD4, CD8, CD45RO, FoxP3, CD56/57, and CD68) were associated with improved prognosis [7]. Activation of the anti-tumor immune response by drugs known as immune checkpoint inhibitors is now being applied to microsatellite instability-high (MSI-H) colorectal tumors due to their high immune reactivity.

Physical activity has been shown to modulate the immune response through a variety of mechanisms. For instance, regular exercise can enhance immune surveillance and reduce chronic inflammation [8]. Furthermore, aerobic fitness was found to correlate with a higher proportion of naive T cells contra senescent T cells in the circulation [9] and an acute bout of exercise may mobilize both NK [10] and CD8 cells to the circulation [11]. In animal models, physical activity has been shown to affect anti-tumor cytotoxicity and both the ratio and absolute numbers of infiltrating cytotoxic T cells in the tumor microenvironment [12, 13]. Even though colon cancer is highly linked to both physical activity and immune infiltration, no previous study has investigated the association between objectively assessed physical activity and immune cell infiltration of the tumor microenvironment in colon cancer.

Most studies examining the relationship between physical activity and cancer outcome rely on self-reported measures of exercise. When using self-reported physical activity, there is always a risk of recall bias, and self-reported physical activity typically overestimates actual physical activity [14]. Physical tests and portable accelerometers offer a more objective and precise means of quantifying physical activity and fitness.

We hypothesized that higher preoperative physical activity and fitness are associated with a higher degree of immune cell infiltration of the tumor microenvironment in colon cancer. We investigated this association using data on both physical activity and fitness collected objectively for the Physical Activity and its impact on COlon cancer Surgery (PACOS) prospective cohort, along with immune cell infiltration of the tumor microenvironment quantified by multiplex immunohistochemistry and multispectral imaging.

## Methods

### Study cohort

This was a prospective cohort study on patients diagnosed with colon cancer with scheduled curative surgery without prior neoadjuvant treatment (stages I–III). The study participants were recruited between January 2019 and February 2024. Due to the COVID19 pandemic, few inclusions were made between March 2020 and April 2021, the only one being in September 2020. The participants were recruited from Umeå University Hospital, Västerbotten Region, Sweden, but were operated on at one of two hospitals: Umeå University Hospital and Skellefteå Hospital; a county hospital in the same region. Two (*n* = 2) participants were referred for surgery in another region due to lack of available hospital beds at the two study hospitals. These two participants were not included due to pathology specimens not being available for analysis. Inclusion criteria in the PACOS study were age > 18 years, newly diagnosed colon cancer, and planned curative surgery (stages I-III), able to speak and understand Swedish, and no neoadjuvant treatment. The participants were recruited either during their preoperative visit to the outpatient clinic at the time of colon cancer diagnosis or by telephone a few days after diagnosis. All participants gave their informed consent to participate in the study after verbal and printed information.

**Fig. 1:**
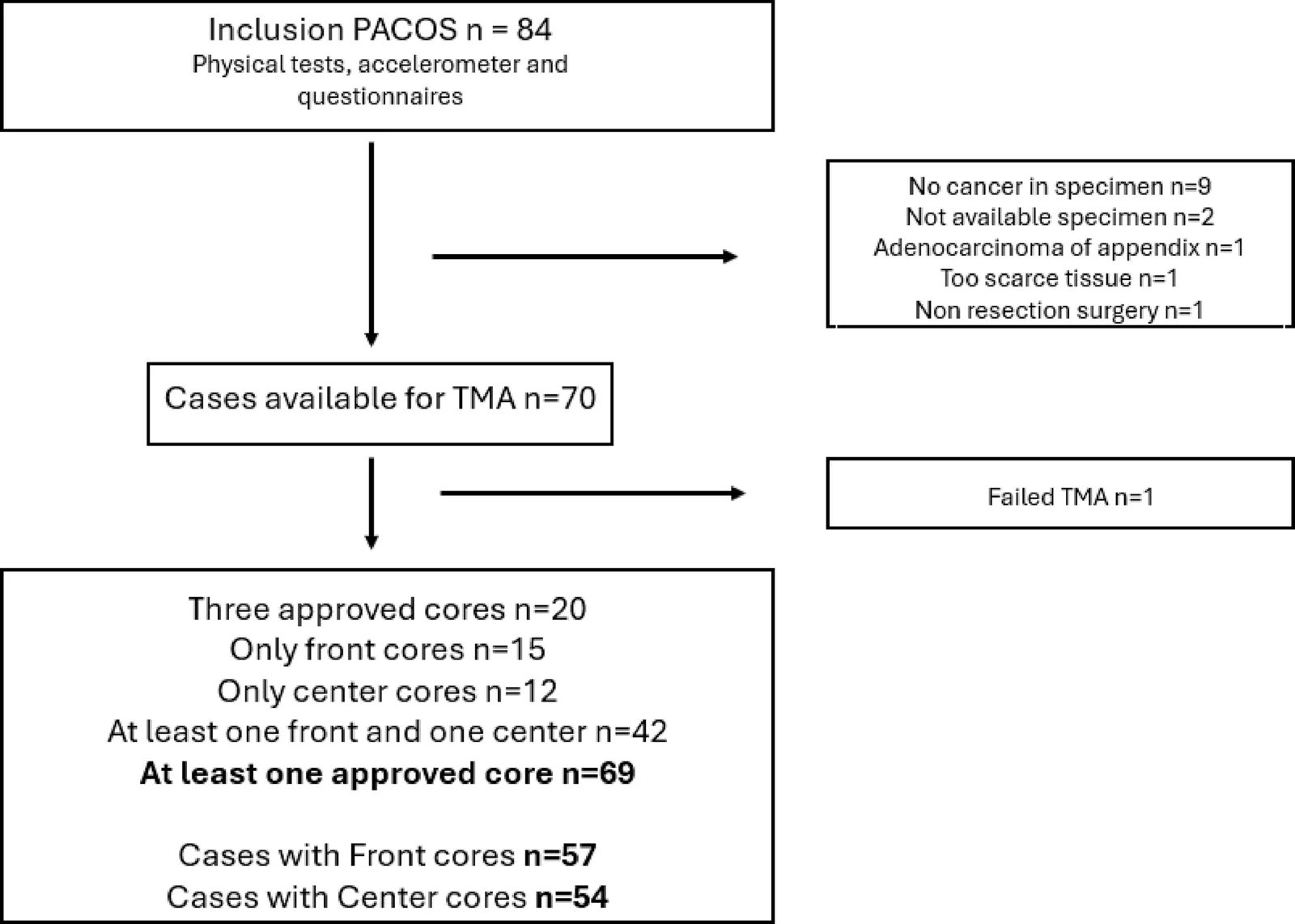
Flow chart of included patients and reasons for exclusion from the original PACOS study population

The present study used a cohort (PACOS) originally collected to investigate the association between physical activity and postoperative complications following colon cancer surgery. In the PACOS study, 84 participants were included where one (*n* = 1) was excluded due to no resection surgery. After further exclusions, 70 specimens available for immune cell infiltration analysis (Fig. 1).

### Variables collected in the PACOS study

On inclusion in the PACOS study, participants were scheduled for a preoperative visit to the clinical research facility at Umeå University hospital for the conduction of physical function tests, to fill in questionnaires and be fitted with an accelerometer.

Physical function tests included the 2-min-step test (2MST), 30-s sit-to-stand test (30STS test), and test of handgrip strength using a handheld dynamometer. The 2MST measures aerobic endurance by calculating the number of times the right leg is raised to a specific mark halfway between the height of the iliac crest and the patella over 2 min. The 30STS test reflects lower body strength and balance and counts the number of times a person can rise from a seated position with their arms across the chest. For both the 2MST and 30STS test, normative values are available based on 2140 individuals aged 60 years and above [15]. The test of handgrip strength was performed using a handheld dynamometer (Saehan Hand Dynamometer SH5001). The participants were instructed to perform two maximal compressions with their dominant hand. The best result of the two was chosen. Handgrip strength correlates well with general muscle strength [16].

Physical activity and sedentary time were derived from data recorded by the accelerometer. A triaxial accelerometer was used (ActiGraph model GT3XPlus, ActiGraph, Pensacola, FL, USA). Participants carried the accelerometer on an elastic belt over the right hip during awake hours for seven consecutive days. In some cases, the time between the visit to the clinical research facility and scheduled surgery was less than 7 days. In these cases, the participants wore the accelerometer for as many days as possible. Accelerometer data were downloaded and processed using Actilife V6.13.5 (ActiGraph, Pensacola, FL, USA). The raw data from three axes were extracted as 60-s epochs. Non-recorded time was defined as > 90 min without movement but with acceptance of a 2-min interval of non-zero counts given that there were zero counts during the 30 min before and after. Only actual wear time was included in the analyses. Participants with a minimum of 600 min of valid time over a minimum of 4 days were included [17]. For participants without the possibility of four days wear time due to scheduled surgery, three consecutive days of 600-min recording time were accepted. Activations in any of the three accelerometer axes were referred to as a count; the higher the count the higher the physical activity. Sedentary time was defined as < 200 counts per minute. Light physical activity as 200–2689, moderate physical activity as 2690–6166, vigorous physical activity as 6167–9642, and very vigorous as > 9643 counts per minute [18]. Moderate to vigorous activity (MVPA) was defined as above 2690 counts per minute.

All participants underwent a whole-body dual-energy X-ray absorptiometry (DXA) scan to measure body composition using a Lunar DPX series iDXA QC Phantom® (GE Healthcare, Madison, WI, USA). The appendicular skeletal muscle mass (AMM) was assessed by adding the sum of the lean mass in grams from the four extremities. Appendicular skeletal mass index (ASMI) was calculated as AMM/height^2^ [16]. Further variables collected at the clinical research facility center were age, height, length, body mass index (BMI), and sex. Clinical variables collected from the medical records were surgical method, length of stay (days), operation time (minutes), tumor location, postoperative complication (yes/no with Clavien-Dindo classification), intraoperative bleeding (mL), American Society of Anesthesiologists (ASA) class, tumor node metastasis (TNM) classification, and stage. Variables from preoperative blood samples were also obtained from the medical records and included, C-reactive protein (CRP) (mg/L), hemoglobin (g/L), white blood cell count (10^9^/L), creatinine (µmol/L), albumin (g/L), and carcinoembryonic antigen (µg/L).

### Multispectral imaging for immune cell infiltration

A tumor tissue microarray (TMA) was prepared including all available tumor specimens (*n* = 70). The TMA blocks were constructed using a TMA GRAND Master Instrument (3DHISTEC, Budapest, Hungary) by punching 1-mm cores from formalin-fixed paraffin-embedded tissue samples. The TMA used included three cores from each specimen, two taken from the invasive tumor front and one taken in the tumor center. The tumor areas used were chosen by a consultant gastrointestinal pathologist.

Immune cell infiltration was evaluated using the Vectra 3® system for multispectral quantitative automated pathology imaging (Akoya Biosciences), as performed previously by this research group and described previously [19]. In short, the TMA was sectioned and mounted onto slides, then immunohistochemically stained using antibodies against Tbet (T-helper 1 cells (Th1)), CD8 (cytotoxic T cells), CD20 (B cells), FoxP3 (regulatory T cells), CD68 (macrophages), and pan-cytokeratin. The imaged slides were imported to the software InForm® (Akoya Biosciences). After the staining and imaging procedures, 187 cores (89%) from 70 patients were available for further immune cell analysis. In total, six specimens had one front core missing, five specimens had both front cores missing, one specimen had both one front core and one center core missing, and five specimens had center cores missing.

In order to quantify immune cell infiltration, the InForm® algorithm was trained on 15 TMA cores to perform tissue segmentation, cell segmentation, and cell phenotyping [20]. The trained algorithm was then used on all TMA specimens.

The image analysis was supervised by an experienced consultant gastrointestinal pathologist (RP) and all exclusions were double-checked before removed from the data set. All investigators were blinded to patient data. Reasons for TMA core exclusion were a large part or the whole core lost (*n* = 9), tumor or stromal tissue missing (*n* = 30), poor staining quality (*n* = 8), or large areas with necrosis (*n* = 3). After TMA core exclusions, 137 (65%) of the original TMA cores were included for analysis. Of the 70 specimens included, one (*n* = 1) had all their cores excluded and the final cohort consisted of 69 patients, of whom 42 (61%) had acceptable cores from both invasive front and center areas. In all, 57 (83%) cases had one or two front cores and 54 (78%) had a center core (Fig. 1).

**Fig. 2:**
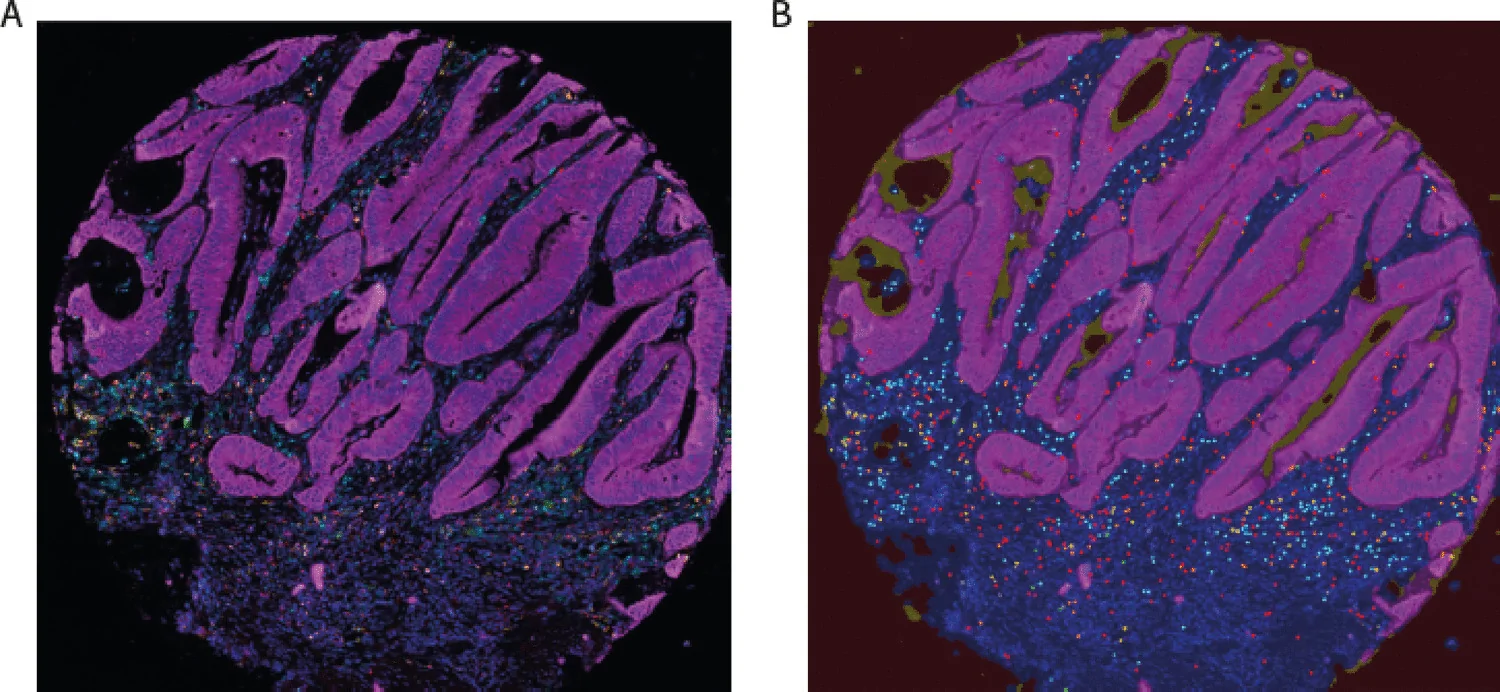
Multispectral imaging of immune cell infiltration in the invasive tumor front of a colon cancer study patient. Shown are high-magnification areas (20×) of a representative multiplex immunohistochemistry stain. **A** Displays the multiplex image after spectral unmixing, with cytokeratin (magenta), CD8+ cytotoxic T cells (red), Tbet+ Th1 cells (green), FoxP3+ T regulatory cells (orange), CD20+ B cells (yellow), and CD68+ macrophages (cyan). **B** Displays the same multiplex immunohistochemistry with tissue segmentation map including stromal area (blue), tumor epithelial area (magenta), tumor debris (yellow) and no tissue (brown), and the cell phenotypes highlighted using the above-described colors

Data on immune cell infiltration were registered as cells per mm^2^ for both the stromal and intraepithelial compartment for respective cell types in both the invasive tumor front and center cores (Fig. 2). When two front cores were available for analysis, the number of cells and tissue areas was added and the cells per mm^2^ were calculated from the sum.

### Exposures and outcomes

The main exposure variable in this study was minutes in MVPA from accelerometer. Other variables collected from physical function tests were number of leg lifts in the 2MST, number repetitions in the 30STS test, and the handgrip force developed in Newton meters (Nm). Accelerometer data were dichotomized into high or low based on whether they achieved the MVPA time recommended by the World Health Organization [21].

A variable based on a combination of the physical function tests was designed. To be grouped into this variable, the participant needed to exceed the sex-specific median handgrip strength, and above age-stratified median values for both the 2MST and the 30STS test. Participants were considered missing if they had missing values in any of three physical functioning tests.

Immune cell infiltration outcomes were reported as cells per mm^2^ for each cell type (CD8^+^, Tbet^+^, CD20^+^, CD68^+^, and FoxP3^+^) in both invasive tumor front and tumor center. Immune cell infiltration values for each cell type were also dichotomized into high and low, with the two lowest tertiles defined as “low” and the highest tertile as “high.”

### Statistics

Normal distribution was checked graphically. When comparing two groups with normally distributed variables, a Student’s *t*-test was used. When the assumption of normal distribution failed, the Mann-Whitney *U* test was used. Assessment of association between physical activity/fitness and immune cell infiltration was made using univariate logistic regression with the immune cell infiltration (high/low) for each cell type in each location as the dependent variable with physical activity/fitness as the independent variable. Multivariable models adjusting for the covariates sex and age were also conducted. Missing data was handled using listwise deletion. Adjustment for multiple testing was made using Bonferroni-corrected *p*-values as well as the Benjamini-Hochberg method.

All statistical analyses were conducted using STATA version 18.0 (StataCorp LP). No prespecified statistical analysis plan was used. This study was reported following the STROBE guidelines (Supplementary Table 1).

This study was approved by the Regional Ethics Committee in Umeå (Dnr 2018-52−31, 2018-299−32, 2018-333−32, 2019–03091) and an amendment was approved by the Swedish Ethics Review Authority (Dnr 2023-00146−02) and registered at clinicaltrials.gov (ID NCT03947840), registration date 2019-05−09. Other clinical trial numbers are not available.

## Results

The final study population included 69 individuals (Fig. 1). All individuals had accelerometer data but only 61 (88%) had accelerometer data with accepted wear time. Sixty-two (90%) individuals had 2MST data, 67 (97%) had 30STS test data, 68 (99%) had handgrip data, and 69 (100%) had data on body composition. The distribution of clinical variables and the results from the physical tests are described in Table 1.

**Table 1:** Patient characteristics

Patient characteristics	*n *= 69
Age (years)^a^	70.3 (39–88)
Sex, *n* (%)	
Male	44 (63.8)
Female	25 (36.2)
BMI (kg/m^2^)^a^	26.4 (16.4–35.5)
Hb (g/l)^1^	128.8 (85–166)
CRP (mg/l)^1^	7.2 (0.6–58)
Diabetes, *n* (%)	
Yes	10 (14.5)
No	59 (85.5)
Hypertension, *n* (%)	
Yes	40 (58.0)
No	29 (42.0)
Smoker, *n* (%)	
Yes	1 (1.4)
No	38 (55.1)
Previous	30 (56.5)
Stage, *n* (%)	
I	14 (20.6)
II	31 (45.6)
III	23 (33.8)
Missing	1
Tumor Location, *n* (%)	
Right	34 (49.3)
Left	35 (50.7)
Average MVPA per day (minutes)^a^	38.0 (0.7–179.7)
Too little wear time (*n*)	8
Sit-to-stand 30-s (n repetitions)^a^	16.2 (5–29)
Missing (*n*)	2
2-minute step test (n steps)^a^	98.1 (52–159)
Missing (*n*)	7
Hand grip strength (Nm)^a^	37.2 (14–60)
Missing (*n*)	1
ASMI (kg/m^2^)^a^	7.2 (3.8–9.5)

Baseline, clinical variables and physical tests or the final study population

^a^Numbers presented as mean (range)

*ASMI*, appendicular skeletal muscle index; *BMI*, body mass index; *CRP*, C-reactive protein; *Hb*, hemoglobin; *Nm*, Newton meter; *MVPA*, moderate to vigorous activity

The association between immune cell density and clinical parameters is shown in Tables 2 and 3 for invasive tumor front and center respectively. Degrees of association differed somewhat between invasive tumor front and center samples with higher immune cell density in the invasive tumor front in women (Table 2) and in the tumor center in right-sided cancers (Table 3). In both the invasive tumor front and center cores, there was a tendency towards higher immune cell density in the stroma of lower stage tumors, in particular cytotoxic CD8^+^ T cells, CD20^+^ B cells, and CD68^+^ macrophages (Tables 2 and 3).

**Table 2:** Immune cell density in tumor invasive front cores categorized by clinical variables

Front	*N*	CD8^+^	Tbet^+^	FoxP3^+^	CD20^+^	CD68^+^	CD8^+^	Tbet^+^	CD68^+^
Stromal compartment							Intraepithelial compartment		
Sex									
Male	36	180 (86–350)	36 (12–60)	23 (6–49)	9 (1–30)	286 (184–518)	15 (3–52)	0.89 (0–7.5)	10 (3–24)
Female	21	341 (206–503)	39 (19–50)	32 (6–63)	36 (8–124)	487 (339–809)	14 (3–56)	1.07 (0–4.7)	11 (0–25)
Age									
< 59	12	220 (101–403)	43 (15–63)	23 (3–58)	19 (8–89)	358 (178–787)	39 (6–82)	2.3 (0–11.5)	8 (0–30)
60–69	11	224 (72–410)	27 (9–34)	13 (4–63)	27 (5–103)	564 (201–817)	13 (3–45)	0.74 (0–5.1)	15 (2–26)
70–79	21	291 (130–466)	39 (14–72)	22 (9–57)	14 (5–63)	321 (241–408)	14 (0–50)	0.84 (0–7.2)	13 (5–18)
>80	13	182 (87–341)	39 (4–60)	32 (22–54)	0 (0–9)	288 (174–525)	12 (6–82)	1.9 (0–4.7)	9 (2–23)
Location									
Left	30	229 (106–343)	28 (15–50)	19 (6–63)	23 (6–66)	386 (267–646)	14 (4–56)	0.4 (0–7.5)	12 (3–26)
Right	27	206 (115–587)	46 (11–70)	30 (5–54)	8 (0–58)	318 (174–632)	16 (0–55)	1.9 (0–5.2)	10 (2–23)
Stage									
I	11	361 (156–466)	39 (10–70)	22 (9–98)	41 (8–148)	408 (201–809)	50 (3–57)	0.9 (0–5.6)	15 (6–25)
II	25	324 (115–410)	28 (11–65)	23 (5–58)	9 (3–46)	339 (223–623)	12 (0–72)	0 (0–7.5)	5 (0–24)
III	20	163 (99–226)	38 (16–52)	29 (8–49)	12 (1–44)	295 (176–459)	14 (6–41)	2.9 (0–6.3)	15 (5–24)
Missing	1								

Clinical variables according to immune cell density in the tumor invasive front. Numbers of cells expressed as median of cells per mm^2^ (25th–75th percentile)

*CD8*^*+*^, cytotoxic T cells; *CD20*^*+*^, B cells; *CD68*^*+*^, macrophages; *Tbet*^*+*^, T-helper 1 cells; *FoxP3*^*+*^, regulatory T cells

**Table 3:** Immune cell density in tumor center cores categorized by clinical variables

Center	*N*	CD8^+^	Tbet^+^	FoxP3^+^	CD20^+^	CD68^+^	CD8^+^	Tbet^+^	CD68^+^
Stromal compartment							Intraepithelial compartment		
Sex									
Male	35	217 (77–381)	19 (10–45)	24 (7–68)	6 (0–18)	469 (254–711)	10 (6–39)	0 (0–4.4)	11 (4–31)
Female	19	103 (43–216)	12 (3–41)	40 (8–89)	7 (0–23)	347 (216–500)	7 (0–44)	0 (0–2.4)	3 (0–10)
Age									
< 59	9	97 (77–192)	10 (6–21)	24 (16–66)	10 (8–16)	465 (329–711)	12 (6–39)	0 (0–2.4)	7 (0–55)
60–69	11	96 (41–381)	14 (3–25)	14 (0–75)	9 (0–23)	402 (201–920)	6 (3–42)	0 (0–1.5)	5 (0–19)
70–79	22	122 (66–255)	14 (7–26)	24 (7–68)	3 (0–18)	377 (229–509)	9 (3–21)	0 (0–2.4)	5 (2–14)
>80	12	208 (130–316)	48 (15–94)	41 (16–69)	7 (0–40)	485 (236–630)	15 (6–62)	1.3 (0–9.0)	12 (7–39)
Location									
Left	27	96 (55–192)	10 (3–19)	20 (8–68)	9 (0–18)	266 (201–465)	6 (3–16)	0 (0–2.4)	4 (0–8)
Right	27	226 (114–429)	23 (15–98)	31 (7–89)	5 (0–19)	488 (395–711)	17 (8–61)	0 (0–6.0)	12 (6–28)
Stage									
I	10	207 (29–691)	14 (3–22)	19 (5–40)	12 (0–18)	547 (61–920)	9 (0–68)	1.6 (0–6.0)	6 (0–19)
II	24	157 (79–316)	21 (10–69)	22 (9–69)	9 (0–19)	453 (269–720)	14 (7–34)	0 (0–4.7)	6 (4–34)
III	19	111 (74–242)	14 (6–41)	49 (11–92)	4 (0–16)	347 (223–488)	8 (4–21)	0 (0–2.1)	7 (0–20)
Missing	1								

Clinical variables according to immune cell density in the tumor center. Numbers of cells expressed as median of cells per mm^2^ (25th–75th percentile)

*CD8*^*+*^, cytotoxic T cells; *CD20*^*+*^, B cells; *CD68*^*+*^, macrophages; *Tbet*^*+*^, T-helper 1 cells; *FoxP3*^*+*^, regulatory T cells

When analyzing the group with accelerometer data and accepted wear time, the group that did not meet the WHO recommendations was older, more anemic, higher rate of diabetes, and more often had right-sided cancer (Table 4).

**Table 4:** Variables according to accelerometer data

MVPA recommendations	*n*	Under *n *= 24	Over *n *= 37	Non-wear^a^ *n *= 8
Sex, *n* (%)				
Male	44	14 (58)	23 (62)	7
Female	25	10 (42)	14 (38)	1
Age, median (25–75^th^ percentile)	69	77.5 (74–81)	68 (59–77)	79.5 (58–82)
BMI, mean (SD)	69	25.8 (4)	27.0 (4)	25.4 (5)
Smoker, *n* (%)				
Yes	1	0 (0)	1 (3)	0
No	38	12 (50)	21 (57)	5
Previous	30	12 (50)	15 (41)	3
Tumor location, *n* (%)				
Right	34	16 (67)	14 (38)	4
Left	35	8 (33)	23 (62)	4
Stage, *n* (%)				
I	14	7 (29)	6 (16)	1
II	31	11 (46)	18 (49)	2
III	23	6 (25)	13 (35)	4
Missing	1			1
Hb preoperative, mean (SD)	69	120 (15)	135 (14)	124 (19)
CRP preoperative, median (25–75th percentile)	69	4.4 (1.7–11.5)	3.1 (1.2–4.8)	4.9 (1.9–13.5)
Diabetes, *n* (%)				
Yes	10	7 (29)	2 (5)	1
No	59	17 (71)	35 (96)	7
Hypertension, *n* (%)				
Yes	40	16 (67)	23 (62)	1
No	29	8 (33)	14 (38)	7

Baseline and clinical variables according to moderate to vigorous activity (MVPA) per day from accelerometer data in the final data set of 69 participants. Missing categories and non-wear column not included in the statistics calculations

^a^Did not wear the accelerometer the predefined amount of time (observations registered as missing data)

*BMI*, body mass index; *CRP*, C-reactive protein; *Hb*, hemoglobin; *MVPA*, moderate to vigorous activity; *SD*, standard deviation

Associations between higher number of leg lifts in the 2MST and higher ASMI and lower immune cell density were evident for both cytotoxic CD8^+^ T cells and CD20^+^ B cells in the stromal compartment of the invasive tumor front cores (Table 5). However, when correcting for multiple testing, these associations were not significant.

**Table 5:** Immune cell infiltration in the invasive tumor front

Immune cell density in tumor front	*n*	*n*	Accelerometer^c^	*n*	2-min step test^d^	*n*	30-s sit-to-stand^e^	*n*	Hand grip^f^	*n*	ASMI^g^
Stromal compartment											
CD8^+^											
Low	38	30	31 (2.8–38.3)	33	103 (18)	37	16 (5)	37	37 (11)	38	7.4 (1.0)
High	19	19	19 (7.5–48.8)	18	89 (30)	19	15 (6)	19	34 (11)	19	6.6 (1.1)
Missing		8		6		1		1			
*p*-value			0.461^a^		0.044^b^		0.477^b^		0.335^b^		0.006^b^
Tbet^+^											
Low	38	31	25 (14.4–57.8)	35	98 (21)	38	17 (6)	38	36 (10)	38	7.0 (1.0)
High	19	18	14 (1.7–60.9)	16	96 (29)	18	15 (5)	18	37 (14)	19	7.3 (1.1)
Missing		8		6		1		1			
*p*-value			0.357^a^		0.784^b^		0.148^b^		0.830^b^		0.341^b^
FoxP3^+^											
Low	38	33	23 (10.3–40)	33	98 (22)	37	16 (5)	38	37 (10)	38	7.2 (1.0)
High	19	16	34 (2.2–69.4)	18	96 (27)	19	17 (6)	18	35 (14)	19	7.0 (1.2)
Missing		8		6		1		1			
*p*-value			0.796^a^		0.787^b^		0.613^b^		0.539^b^		0.600^b^
CD20^+^											
Low	38	33	22 (8.6–57.8)	34	102 (23)	37	16 (6)	38	38 (11)	38	7.3 (1.0)
High	19	16	34 (3.5–54.5)	17	88 (23)	19	16 (5)	18	33 (12)	19	6.7 (1.2)
Missing		8		6		1		1			
*p*-value			0.780^a^		0.046^b^		0.723^b^		0.168^b^		0.042^b^
CD68^+^											
Low	38	32	23 (11.1–39.2)	33	96 (19)	37	15 (5)	37	37 (11)	38	7.3 (1.0)
High	19	17	38 (3.5–76)	18	100 (31)	19	17 (7)	19	36 (12)	19	6.8 (1.3)
Missing		8		6		1		1			
*p*-value			0.529^a^		0.606^b^		0.286^b^		0.715^b^		0.174^b^
Intraepithelial compartment											
CD8^+^											
Low	3819	31	25 (10.3–60.9)	34	102 (19)	38	16 (5)	37	36 (11)	38	7.3 (1.0)
High		18	23 (4.2–57.8)	17	90 (30)	18	15 (6)	19	36 (12)	19	6.8 (1.2)
Missing		8		6		1		1			
*p*-value			0.841^a^		0.093^b^		0.505^b^		0.936^b^		0.170^b^
Tbet^+^											
Low	38	33	23 (12–43.8)	34	97 (23)	37	16 (6)	38	36 (11)	38	7.1 (0.9)
High	19	16	28 (3.2–69.6)	17	98 (25)	19	16 (4)	18	37 (12)	19	7.2 (1.4)
Missing		8		6		1		1			
*p*-value			0.996^a^		0.904^b^		0.959^b^		0.715^b^		0.837^b^
CD68^+^											
Low	39	31	22 (10.3–38.3)	35	97 (22)	38	16 (6)	37	36 (10)	38	7.2 (1.0)
High	18	18	39 (3.5–62.8)	16	98 (27)	18	16 (6)	19	37 (13)	19	7.0 (1.3)
Missing		8		6		1		1			
*p*-value			0.431^a^		0.926^b^		0.799^b^		0.725^b^		0.614^b^

Immune cell infiltration in the tumor front divided into low (lowest two tertiles) and high (highest tertile)

^a^Mann-Whitney *U* test

^b^Student *t*-test

^c^Accelerometer data presented as minutes of moderate to vigorous activity (MVPA) per day. Median (25th–75th percentile)

^d^2-min-step test presented as number of leg raises in two minutes. Mean (SD)

^e^30-s sit-to-stand test presented as number of chair raises in 30 s. Mean (SD)

^f^Handgrip strength presented as developed force in nM. Mean (SD)

^g^ASMI (appendicular skeletal muscle index) presented as appendicular muscle mass/height^2^. Mean (SD)

*CD8*^*+*^, cytotoxic T-cells; *CD20*^*+*^, B cells; *CD68*^*+*^, macrophages; *Tbet*^*+*^, T-helper 1 cells; *FoxP3*^*+*^, regulatory T cells

In the tumor center, higher MVPA per day and higher number of repetitions in the 30STS test were associated with lower density of Tbet^+^ Th1 cells in the stromal compartment (Table 6). There was also a non-significant trend towards a higher number of lifts in the 2MST with lower Tbet^+^ Th1 cells in the tumor center (Table 6). However, when correcting for multiple testing, these associations were not significant.

**Table 6:** Immune cell infiltration in the tumor center

Immune cell density in tumor center	*n*	*n*	Accelerometer^c^	*n*	2-min step test^d^	*n*	30-s Sit-to- stand^e^	*n*	Hand grip^f^	*n*	ASMI^g^
Stromal compartment											
CD8^+^											
Low	37	30	28 (8.2–50.3)	33	99 (21)	35	17 (6)	37	36 (11)	37	7.0 (1.2)
High	17	16	31 (10.4–59.6)	15	92 (26)	17	15 (6)	16	41 (12)	17	7.3 (1.1)
Missing		8		6		2		1			
*p*-value			0.868^a^		0.345^b^		0.416^b^		0.111^b^		0.497^b^
Tbet^+^											
Low	36	31	38 (21.7–60.9)	34	101 (19)	35	18 (5)	36	38 (11)	36	7.2 (1.1)
High	18	15	6 (2–48.8)	14	88 (28)	17	13 (5)	17	35 (12)	18	7.0 (1.2)
Missing		8		6		2		1			
*p*-value			0.023^a^		0.076^b^		0.005^b^		0.284^b^		0.510^b^
FoxP3^+^											
Low	36	30	31 (8.6–50.3)	32	97 (22)	36	16 (6)	35	37 (10)	36	7.0 (1.1)
High	18	16	28 (2.4–64.2)	16	97 (25)	16	16 (6)	18	37 (14)	18	7.3 (1.2)
Missing		8		6		2		1			
*p*-value			0.914^a^		0.951^b^		0.610^b^		0.810^b^		0.380^b^
CD20^+^											
Low	36	32	28 (8.4–64.0)	32	98 (23)	35	16 (5)	35	37 (12)	36	7.1 (1.2)
High	18	14	31 (4.5–48.0)	16	96 (24)	17	16 (7)	18	38 (11)	18	7.1 (1.1)
Missing		8		6		2		1			
*p*-value			0.719^a^		0.804^b^		0.759^b^		0.762^b^		0.898^b^
CD68^+^											
Low	36	30	28 (8.6–60.9)	33	98 (18)	36	16 (5)	36	35 (11)	36	7.1 (1.0)
High	18	16	31 (5.3–53.3)	15	95 (31)	16	17 (8)	17	42 (13)	18	7.2 (1.3)
Missing		8		6		2		1			
*p*-value			0.950^a^		0.719^b^		0.711^b^		0.065^b^		0.758^b^
Intraepithelial compartment											
CD8^+^											
Low	36	28	31 (10.1–55.6)	31	100 (20)	35	16 (5)	35	37 (11)	36	7.2 (1.1)
High	18	18	27 (6.4–57.8)	17	91 (27)	17	16 (7)	18	39 (12)	18	6.9 (1.3)
Missing		8		6		2		1			
*p*-value			0.898^a^		0.184^b^		0.921^b^		0.549^b^		0.319^b^
Tbet^+^											
Low	36	30	35 (12.0–60.9)	33	96 (20)	34	16 (6)	35	37 (12)	36	7.1 (1.3)
High	18	16	24 (2.8–53.3)	15	99 (29)	18	16 (6)	18	38 (11)	18	7.2 (0.8)
Missing		8		6		2		1			
*p*-value			0.351^a^		0.633^b^		0.761^b^		0.652^b^		0.674^b^
CD68^+^											
Low	37	31	38 (12.0–60.9)	34	98 (22)	37	16 (6)	37	36 (11)	37	7.0 (1.0)
High	17	15	16 (2.8–57.8)	14	96 (25)	15	16 (6)	16	40 (12)	17	7.3 (1.4)
Missing		8		6		2		1			
*p*-value			0.238^a^		0.766^b^		0.844^b^		0.321^b^		0.320^b^

Immune cell infiltration in the tumor center divided into low (lowest two tertiles) and high (highest tertile)

^a^Mann-Whitney *U* test

^b^Student *t*-test

^c^Accelerometer data presented as minutes of moderate to vigorous activity (MVPA) per day. Median (25th–75th percentile)

^d^2-min-step test presented as number of leg raises in two minutes. Mean (SD)

^e^30-s sit-to-stand test presented as number of chair raises in 30 s. Mean (SD)

^f^Handgrip strength presented as developed force in nM. Mean (SD)

^g^ASMI (appendicular skeletal muscle index) presented as appendicular muscle mass/height^2^. Mean (SD)

*CD8*^*+*^, cytotoxic T cells; *CD20*^*+*^, B cells; *CD68*^*+*^, macrophages; *Tbet*^*+*^, T-helper 1 cells; *FoxP3*^*+*^, regulatory T cells

In the logistic regression models, a better result in the 30STS test was associated with lower density of Tbet^+^ Th1 cells in the stromal compartment of the tumor center (Table 7). When correcting for multiple testing, this result was no longer significant. In the invasive tumor front samples, no significant association was seen in the models adjusted for age and sex, but there was a non-significant association between higher ASMI and a lower density of cytotoxic CD8^+^ T cells (Table 7). A high combined fitness value was not significantly associated with increased immune cell density in the invasive tumor front. Results were more scattered in the tumor center cores and varied from negative ORs for cytotoxic CD8^+^ T cells and FoxP3^+^ regulatory T cells to positive ORs around 3 for CD20^+^ B cells and CD68^+^ macrophages. All analyses had wide confidence intervals, and no results were significant (Table 7).

**Table 7:** Logistic regression of association between immune cell density and physical activity/fitness

**Invasive front**	***N***	**CD8** ^**+**^	**Tbet** ^**+**^	**FoxP3** ^**+**^	**CD20** ^**+**^	**CD68** ^**+**^
**Stromal compartment**						
Accelerometer						
Univariable	49	0.99 (0.98–1.01)	1.00 (0.98–1.01)	1.00 (0.99–1.02)	1.00 (0.98–1.01)	1.01 (0.99–1.02)
Multivariable	49	0.99 (0.98–1.01)	1.00 (0.98–1.02)	1.01 (0.99–1.03)	1.00 (0.98–1.02)	1.01 (0.99–1.03)
2-min step test						
Univariable	51	0.97 (0.95–1.00)	1.00 (0.97–1.02)	1.00 (0.97–1.02)	0.97 (0.94–1.00)	1.01 (0.98–1.03)
Multivariable	51	0.97 (0.94–1.01)	1.00 (0.97–1.03)	1.01 (0.98–1.04)	0.96 (0.93–1.00)	1.01 (0.98–1.04)
Sit-to-stand test						
Univariable	56	0.96 (0.87–1.07)	0.92 (0.82–1.03)	1.03 (0.93–1.14)	0.98 (0.89–1.09)	1.06 (0.95–1.17)
Multivariable	56	0.98 (0.87–1.10)	0.92 (0.81–1.04)	1.05 (0.94–1.17)	0.98 (0.87–1.10)	1.06 (0.95–1.19)
Hand strength						
Univariable	56	0.97 (0.93–1.03)	1.01 (0.96–1.06)	0.98 (0.93–1.04)	0.96 (0.91–1.02)	0.99 (0.94–1.04)
Multivariable	56	1.09 (0.96–1.19)	1.00 (0.91–1.10)	1.07 (0.96–1.19)	1.03 (0.92–1.15)	1.03 (0.93–1.14)
Combined Fit						
Univariable	50	0.86 (0.22–3.37)	1.08 (0.27–4.31)	0.86 (0.22–3.37)	1.55 (0.41–5.89)	1.37 (0.36–5.19)
Multivariable	50	1.01 (0.26–4.55)	1.17 (0.28–4.89)	1.03 (0.25–4.28)	1.84 (0.43–7.82)	1.41 (0.35–5.65)
ASMI						
Univariable	57	0.45 (0.24–0.84)^a^	1.30 (0.76–2.23)	0.87 (0.52–1.45)	0.57 (0.32–1.00)	0.69 (0.41–1.18)
Multivariable	57	0.47 (0.22–1.01)	1.42 (0.71–2.84)	1.15 (0.59–2.25)	0.65 (0.32–1.33)	0.69 (0.35–1.35)
**Center**	***N***	**CD8** ^**+**^	**Tbet** ^**+**^	**FoxP3** ^**+**^	**CD20** ^**+**^	**CD68** ^**+**^
**Stromal compartment**						
Accelerometer						
Univariable	46	1.00 (0.98–1.01)	0.99 (0.97–1.01)	1.00 (0.99–1.02)	1.00 (0.98–1.02)	1.00 (0.99–1.02)
Multivariable	46	1.00 (0.98–1.02)	0.99 (0.97–1.01)	1.00 (0.98–1.02)	1.00 (0.98–1.02)	1.00 (0.98–1.02)
2-min step test						
Univariable	48	0.99 (0.96–1.01)	0.97 (0.94–1.00)	1.00 (0.97–1.03)	1.00 (0.97–1.02)	0.99 (0.97–1.02)
Multivariable	48	0.98 (0.95–1.01)	0.97 (0.94–1.01)	1.00 (0.97–1.03)	1.00 (0.96–1.02)	0.99 (0.96–1.02)
Sit-to-stand test						
Univariable	52	0.96 (0.86–1.06)	0.83 (0.72–0.96)^1^	0.97 (0.87–1.08)	1.02 (0.92–1.13)	1.02 (0.92–1.13)
Multivariable	52	0.92 (0.81–1.05)	0.82 (0.70–0.96)^1^	0.98 (0.87–1.10)	1.01 (0.90–1.14)	0.99 (0.88–1.12)
Hand strength						
Univariable	53	1.04 (0.99–1.10)	0.97 (0.92–1.02)	0.99 (0.95–1.04)	1.01 (0.96–1.06)	1.05 (1.00–1.11)
Multivariable	53	1.09 (0.97–1.22)	0.96 (0.86–1.06)	0.99 (0.90–1.10)	1.05 (0.94–1.16)	1.06 (0.95–1.18)
Combined Fit						
Univariable	46	0.17 (0.02–1.53)	0.57 (0.10–3.12)	0.86 (0.19–3.92)	1.73 (0.40–7.47)	1.73 (0.40–7.47)
Multivariable	46	0.13 (0.01–1.28)	0.62 (0.11–3.50)	0.92 (0.20–4.29)	1.70 (0.38–7.53)	1.57 (0.35–6.94)
ASMI						
Univariable	54	1.20 (0.71–2.03)	0.84 (0.50–1.40)	1.27 (0.75–2.13)	1.03 (0.62–1.71)	1.09 (0.65–1.81)
Multivariable	54	0.99 (0.48–2.04)	0.93 (0.45–1.90)	1.71 (0.79–3.69)	1.11 (0.56–2.21)	0.71 (0.35–1.43)

Logistic regression with immune cell infiltration in the invasive tumor front and center as the dependent variable. Multivariable models adjusted for age and sex

^a^Significant finding (*p* < 0.05)

*ASMI*, appendicular skeletal muscle index. *CD8*^*+*^, cytotoxic T cells; *CD20*^*+*^, B cells; *CD68*^*+*^, macrophages; *Tbet*^*+*^, T-helper 1 cells; *FoxP3*^*+*^, regulatory T cells

When analyzing the three physical tests, MVPA per day, and body composition assessed by ASMI, better physical fitness/activity was associated with male sex, higher stage, and left-sided cancer (data not shown).

## Discussion

This study is the first to objectively assess physical activity and fitness and investigate its association with immune cell infiltration in primary colon tumors. Our results did not support the hypothesis of the study that better preoperative physical activity is associated with increased tumor immune cell infiltration.

In both univariable and multivariable models, higher numbers of repetitions in the 30STS test were significantly associated with lower immune cell infiltration of Th1 cells in the stromal compartment of the tumor center. No such association was seen in the invasive tumor front. However, when adjusting for multiple testing, the results were all non-significant and the clinical implications are therefore debatable. In a recent preclinical study on mice with breast cancer the effect of exercise on Th1 cytokine profile was investigated. Exercising mice had significantly more tumor necrosis factor-α (TNF-α) in the tumor tissue (one of the cytokines produced by Th1 cells) though no effect was seen on interferon-γ (IFN-γ), another important cytokine produced by Th1 cells [22]. In another study on twelve human ovarian cancer survivors, resistance exercise four times a week for 12 weeks resulted in an increase in the number of circulating Th1 cells and decreased the number of Th2 cells [23]. The results of the present study differ in that higher fitness score was associated with lower Th1 cell infiltration. However, the studies differ substantially in methodology and no previous study has investigated the association between physical activity/exercise and Th1 cell infiltration in the tumor microenvironment in colorectal cancer patients.

There are limited data on how exercise and physical activity affect tumor infiltration by different immune cell types in animals and humans. Rundqvist et al. showed that exercising mice with mammary cancer had increased tumor infiltration by cytotoxic T cells and that no differences were seen for other cell types analyzed including CD4^+^ T cells, macrophages, natural killer (NK) cells, and neutrophils [12]. In another preclinical mouse breast cancer model, the authors showed that exercise increased the amount of active cytotoxic T cells, defined as CD8^+^/CD69^+^, in the tumor microenvironment. However, the total number of cytotoxic T cells was not significantly different when comparing exercising mice with controls [24]. Kim et al. analyzed the effect of exercise and high-fat diet in mice with mammary cancer and showed a significant decrease in pro-tumor M2 macrophages in mice with high-fat diet and exercise, compared to controls. No difference was seen for anti-tumor M1 macrophages [25]. Another study investigating the effect of exercise and high-fat diet on macrophage density in the colon in mice with induced colon cancer failed to show an association between macrophage infiltration and exercise whereas the high-fat diet increased macrophage density regardless of exercise [26]. Almeida et al. investigated the effect of swimming on tumor-bearing mice and found lower macrophage infiltration in the swimming group compared to sedentary controls [27]. Data on human cancer are even fewer, and there are no intervention studies on exercise as exposure and immune cell infiltration in colorectal cancer. Our research group conducted a retrospective study with prospective self-reported data on recreational physical exercise in colorectal cancer patients and showed higher odds for cytotoxic T cell infiltration in the invasive tumor front and center in individuals exercising more than three times a week [28]. There are some intervention studies with immune cell infiltration in other cancer types as outcome. A previous study on human pancreatic ductal adenocarcinoma showed that preoperative exercise intervention significantly increased the infiltration of cytotoxic CD8^+^ T cells [29]. A Danish research group has studied the effect of exercise intervention on immune cell infiltration of cytotoxic CD8^+^ T cells, CD3^+^ T cells [30], and NK cells [31] in 30 men with prostate cancer. No effect was seen on either cell type in the intention-to-treat analysis, but in the per-protocol analysis, an increase of NK cell infiltration was seen in the intervention group [31]. All previously published intervention studies in this field have small sample sizes, which makes it difficult to draw firm conclusions from. Regarding B cells, no previous preclinical or human study on physical activity and immune infiltration in cancer has been reported to our knowledge. The present study provides a novel insight into infiltration of five different immune cell types in human colon cancer.

The activity of cytotoxic CD8^+^ T cells is highly regulated by both Th1 (Tbet^+^), Th2, and regulatory T cells [32]. When it comes to anti-tumor efficacy, Th1 and cytotoxic T cells are highly linked to each other, and the presence of one of the cell types often predicts the presence of the other [33]. The association of cytotoxic CD8^+^ T cells in the invasive tumor front and Tbet^+^ Th1 in the tumor center with lower physical activity and fitness could reflect a similar response. CD20 is a generic B cell marker, and the role of B cells in anti-tumor pathophysiology is complex, involving the secretion of antibodies and cytokines, and also T cell stimulation [34]. Tumor-infiltrating CD20^+^ B cells have been found in close proximity to cytotoxic CD8^+^ T cells. In ovarian cancer, the beneficial effects of the immune response on survival were enhanced when cytotoxic CD8^+^ T cells were present alongside CD20^+^ B cells rather than when alone [35]. These findings may help explain why the strongest associations in the present study were observed for cytotoxic CD8^+^ T cells, Tbet^+^ Th1, and CD20^+^ B cells. However, the results should be interpreted with caution since this was a study with a low sample size, and there are diverging results between the different physical tests and tumor areas.

Previous studies on body composition and immune infiltration in colorectal cancer are limited. Cheng et al. assessed body composition with computed tomography (CT) and immune infiltration in 52 women with breast cancer and found higher levels of CD8^+^ T cells and lower levels of CD20^+^ B cells in the stromal compartment of tumors in participants with the highest total adipose tissue tertile and the two highest skeletal muscle mass tertiles [36]. Another study on renal cell carcinoma investigated immune infiltration of macrophages, cytotoxic T cells, Th1 cells, Th2 cells, T-helper 17 (Th17) cells, B cells, and regulatory T cells by assessing gene expression patterns and their association with skeletal muscle index (SMI) assessed by CT [37]. Their results showed greater infiltration of macrophages and Th1 cells and less infiltration of Th17 cells with lower SMI. No significant associations were seen for the other cell types [37]. These results differ from those of the present study but so did the study design and cancer type. The results of this study indicate a possible association between high ASMI and lower density of cytotoxic T cells in the invasive tumor front. However, these relationships were not significant when adjusting for age and sex in the logistic regression.

There is evidence supporting higher immune cell infiltration in right-sided tumors, female sex, and lower stage tumors [38, 39]. Previous studies using multiplex immunohistochemistry techniques to assess immune cell infiltration in the tumor microenvironment of colon cancer have shown similar distribution of immune cells in the invasive tumor front and center tumor cores [19, 40]. One possible explanation why immune cell distribution differed between the invasive tumor front and center in our study was the small sample size. In the cohort as a whole, however, the overall results were as expected, with higher immune cell density observed in females, right-sided tumors, and lower stage cancer, indicating that the differences between the front and center cores may not have occurred with a larger sample. The observed tendency towards the expected immune cell distribution suggests that data from the entire cohort constitutes a representative sample of immune cell infiltration.

Studies including objectively assessed data on physical activity and physical fitness are rare in research focusing on immune cell infiltration in colon cancer. Physical activity is difficult to measure and quantify objectively, especially in cancer patients. In this study, we used three different physical function tests and accelerometer readings to assess physical activity levels and fitness. The accelerometer is an often-used and validated tool for measuring physical activity which is why we chose accelerometry as our primary exposure variable. The three different physical fitness tests in the present study differ in the type of fitness they evaluate. The 30STS test evaluates strength and balance in the lower extremities, the 2MST tests aerobic endurance, and the test of handgrip strength tests muscle strength in the upper extremities. Therefore, any one of these fitness tests will not give a full picture of the participant’s fitness level and could explain why the results in this study deviated between the three tests. To compensate for this, we constructed a combined variable derived from all three. The results had wide confidence intervals due to the relatively small sample size making interpretation of the results from the combined fitness variable difficult and should be viewed with caution.

As stated in the introduction to this study, both immune cell infiltration in the tumor microenvironment and physical activity are associated with improved prognosis in colon cancer [3, 7]. This study was not designed to investigate the presence of a causal relationship between physical activity and immune infiltration in colon cancer. Consequently, the results neither confirm nor reject that the beneficial effects of physical activity in colon cancer are mediated through enhanced immune cell infiltration in the tumor microenvironment. With this said, the results of this individual study are not sufficient to alter clinical decision-making, and further studies are therefore needed on the topic.

Limitations of the study include the pilot study design, the small sample size, and missing data from the physical function tests which limited the possibility of constructing a combined physical fitness test variable. When registered at clinicaltrials.gov, the immune cell outcome was not specified and was phrased immunological response around the tumor. This was later decided on since the available antibodies for the Vectra system were uncertain at registration. This could be considered a limitation of the study, although unlikely to have affected the results. The analyses in this study can therefore be considered exploratory, which is why statistical testing was only performed for Tables 5, 6, and 7, while Tables 1, 2, 3, and 4 present descriptive results without hypothesis testing. Another limitation is the lack of data on molecular subtypes in the dataset since this could possibly affect the immune cell infiltration [19]. A strength of this study is the objective tests used to assess physical activity and fitness status combined with detailed data on immune cell tumor infiltration. Another strength is the prospective inclusion and detailed clinical and demographic data. The data in this study were collected and analyzed by the same researchers which enabled control and supervision of all data collection.

## Conclusion

After correction for multiple tests, we found no consistent association between objectively assessed data on physical activity and fitness with immune cell density in colon cancer tumor samples. The present study demonstrates feasibility of conducting research using detailed objective assessment of physical activity and immune cell infiltration. A larger study is needed to more thoroughly investigate any association between physical activity and immune cell infiltration in colon cancer.

## Supplementary Information

Below is the link to the electronic supplementary material.

ESM 1 (DOCX 34.9 KB)

## Data Availability

The data used and analyzed during this study are available from the corresponding author upon reasonable request.
